# The Effect of Emotional Labor on Presenteeism of Chinese Nurses in Tertiary-Level Hospitals: The Mediating Role of Job Burnout

**DOI:** 10.3389/fpubh.2021.733458

**Published:** 2021-09-21

**Authors:** Jia Song, Fang Liu, Xiaowei Li, Zhan Qu, Rongqiang Zhang, Jie Yao

**Affiliations:** ^1^School of Nursing, Shaanxi University of Traditional Chinese Medicine, Xianyang, China; ^2^School of Nursing, Health Science Center, Xi'an Jiaotong University, Xi'an, China; ^3^School of public health, Shaanxi University of Traditional Chinese Medicine, Xianyang, China

**Keywords:** presenteeism, emotional labor, burnout, nurses, China

## Abstract

**Background:** Employees who are physically present but work insufficiently because of illness are deemed as having presenteeism. In the health care setting, the issue has taken on greater importance because of the impairment of the physical and mental health of nurses and the nursing safety of the patients. According to the Job Demand-Resource Model, burnout may link emotional labor with presenteeism. Thus, this study analyzed the role of burnout as a mediating factor between the three types of emotional labor strategies and presenteeism among nurses in tertiary-level hospitals.

**Methods:** A cross-sectional study of 1,038 nurses from six Chinese hospitals was conducted. The questionnaires, including the 14-item emotional labor strategies scale, 22-item Maslach Burnout Inventory scale, 6-item Stanford Presenteeism Scale, and items about demographic characteristics and work-related factors, were used to collect data. A multivariable linear regression was used to predict work-related factors and investigate the correlation of emotional labor, burnout, and presenteeism. The structural equation model was implemented to test the mediating effects of job burnout.

**Results:** The results of the study showed that the average presenteeism score of the participants was 14.18 (4.33), which is higher than in Spanish, Portuguese, and Brazilian nurses. Presenteeism was explained by 22.8% of the variance in the final model in multivariable linear regression (*P* < 0.01). Presenteeism was found to be positively correlated with surface acting, emotionally expressed demands, deep acting, emotional exhaustion, depersonalization, and low personal accomplishment (*P* < 0.01). Notably, presenteeism was negatively correlated with deep acting (*P* < 0.01). In addition, burnout partially mediated the correlation between emotionally expressed demands, deep acting, and presenteeism with a mediatory effect of 24 and 63.31% of the total effect. Burnout completely mediated the association between surface acting and presenteeism, a mediating effect of 86.44% of the total effect.

**Conclusions:** The results of this study suggested that different emotional labor strategies affect presenteeism, either directly or indirectly. Nursing managers should intervene to reduce presenteeism by improving the ability of the nurses to manage emotions, thereby alleviating burnout.

## Introduction

Presenteeism has become a common phenomenon in the workplace which is defined as impaired productivity or performance while ill but still working (1–4). The concept has been of great interest for over two decades. More than 80% of healthcare providers and physicians in England and Norway will attend work despite ill health (5, 6). Furthermore, more than 70% of Danish core workers had gone to the workplace while ill (7). Additionally, one cross-sectional study reported that 74% of Chinese employees must work despite being ill; the frequency of presenteeism is almost once a month (8). Previous studies in Turkey, Korea, and the United States had demonstrated the high prevalence and factors of presenteeism, including among doctors and nurses (9–11). Nurses may experience up to four times as much presenteeism as other health or welfare workers in Sweden (12) because of the apparent characteristics of nursing work, such as shift work, an increased workload, low replaceability, and extended working time (13–16). The presenteeism phenomenon may impair the physical and mental health of nurses (8, 17) and reduce health-related productivity and increased economic costs (18, 19). It has also been shown that presenteeism will compromise patient safety and reduce the work quality of nurses by increasing the risk of patient falls and medication errors and disease transmission (11, 20, 21). Therefore, combating presenteeism behavior may be a key to improve the health productivity of nurses. At present, there is little empirical research on the presenteeism of nurses. This study provided a new way of thinking for nursing managers to reduce the presenteeism of nurses.

Managers have taken notice of the link between emotional labor, burnout, and presenteeism. Nurses provide professional medical services as well as face-to-face communication and listening to the concerns of patients. Nursing is an emotionally labor-intensive profession. Emotional labor refers to individual efforts, plans, management of emotional expression, or bodily display in response to organizational demands in the workplace (22). Job burnout is a mental health injury state in which employees suffer from chronic work-related stress (23). The concept of burnout in three dimensions, including emotional exhaustion, depersonalization, and low personal accomplishment, as proposed by the study of Maslach and Jackson is generally accepted (24). The characteristics of burnout are emotional exhaustion, overextended alienation from the job, and a sense of perceived incompetence in positions (23, 24).

The study of Grandey et al. presented two main emotional labor strategies, namely, surface acting and deep acting; other studies have adopted this approach (25, 26). Surface acting is not just suppression but also the regulation of emotion with an expression that matches the expectations of the organization. Deep acting occurs when employees keep inner feelings consistent with their displayed expression (22). This is an antecedent-focused emotional regulation (reappraisal and situation modification), while surface acting is a response-focused emotion regulation (physiological change and expression suppression) (27). According to the meta-analytic structural model of Kammeyer, deep acting is a positive predictor of job performance, while surface acting predicted burnout negatively (28).

Investigators have recently examined the correlation between emotional labor and job burnout, emotional exhaustion, job-related stress, depression symptoms, customer orientation, and presenteeism (29–33). Previous research also has demonstrated that burnout, job-related anxiety, depression, and emotional labor can all be risk elements for presenteeism (16, 30, 32–34). Based on these discussions, job burnout may have a direct or indirect mediating effect on emotional labor and presenteeism.

Job Demand-Resource Model and Emotional Labor theory are applied to understand the correlation between emotional labor, job burnout, and presenteeism. The Job Demand-Resource Model proposes that job demand is the important antecedent of in-role performance through the mediating effect of the emotional exhaustion component of burnout, i.e., cognitive and emotional fatigue (35–37). Job demands are described as work-related aspects requiring sustained physical or mental effort with specific physical or psychological costs, including high work stress, emotional demands, and undesired working conditions (38). In-role performance is defined as meeting organizational targets and practical functions (35). The existing body of research on high job demands suggests that they are associated with occupational burnout (39) and presenteeism (40). At the same time, emotional display required by the organization is a significant job demand, especially for nurses (41). Based on previous studies and the foundation of the Job Demand-Resource Model, we proposed the hypothesis that emotional labor affects presenteeism through the mediating effect of job burnout.

According to the Emotional Labor theory, emotional labor affects individual and organization levels (42). Moreover, Grandey proposed that emotional labor, involving both surface acting and deep acting, is linked to individual well-being, involving both job satisfaction and job burnout, and organizational well-being, involving both performance and withdrawal behavior (22, 27). In addition, a past study demonstrated how Korean nurses associate emotional labor with presenteeism (30). However, few studies have explored these different emotional regulation strategies, which have other effects.

**Figure 1:**
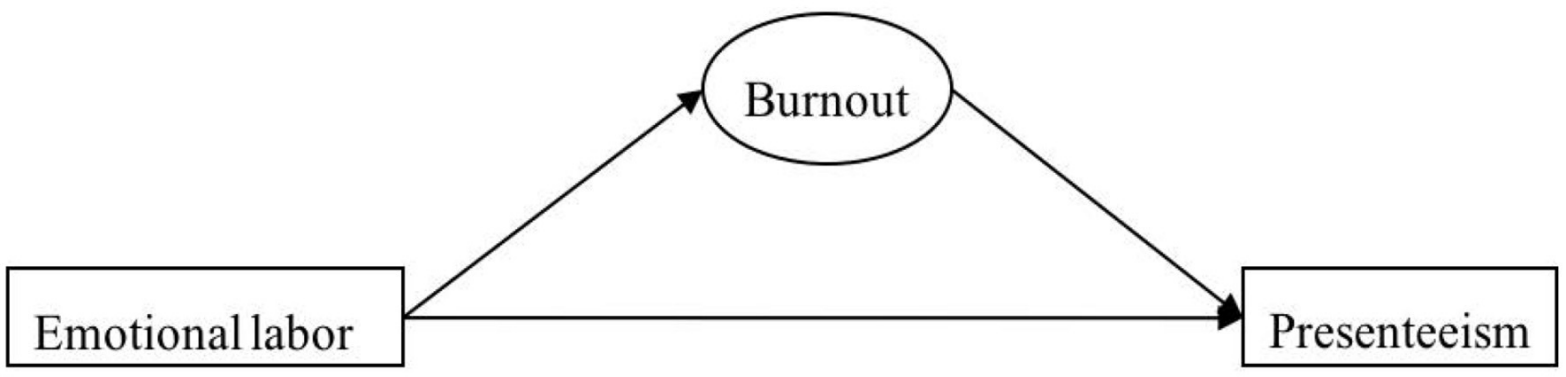
The hypothesized model.

Based on the Job Demand-Resource Model and Emotional Labor Theory, this study examined the correlations between emotional labor, burnout, and presenteeism and the mediating effect of burnout and the relationship between emotional labor and presenteeism in mitigating the low productivity of nurses. It is meaningful to understand how emotional labor affects presenteeism. Figure 1 depicts the hypothesized model.

## Method

### Setting and Sample

Convenience sampling was used to recruit from the six tertiary hospitals in Shaanxi province, China. Six hospitals with similar hospital grades as designated by the Ministry of Health were selected. For a convenience sample, nurses with a professional certificate, informed consent, and voluntary involvement in this study were included. Excluded were nurses who had worked for less than a year and nurses who were on leave for various reasons during the investigation.

The study of Thompson recommended that 10–15 times of questionnaire items count the sample size for the structural equation model (43). The self-administered questionnaire consisted of 42-items, including 14 emotional labor items, 22 job burnout items, and 6 presenteeism items. The total sample size involved 630 participants. However, the sample size was expanded by 20% because of incomplete questionnaires, resulting in a final sample size of 756 participants. A total of 1,054 nurses were recruited.

### Ethical Approval

The nurses voluntarily chose to participate in this study and were free to drop out. The electronic information submitted was anonymous, and only the researchers had access to data. The Ethics Committee of the Affiliated Hospital of Shaanxi University of Traditional Chinese Medicine, Shaanxi Province, China approved this study.

### Data Collection

The study was carried out from October to December 2020. A structured questionnaire with four parts was distributed to all participants, covering sociodemographic information, emotional labor, job burnout, and presenteeism. The participants were volunteers and we assured them that all their information would be kept confidential. Before the questionnaire was issued, we contacted the nursing departments of hospitals, introduced the purpose of this study to obtain permission, and discussed the survey time and the number of respondents. Two trained graduate students went to the six hospitals and conducted the field survey from October to December 2020. The nurses were informed of the purpose, significance, and independent completion of the survey. The questionnaires were completed on-site with the participation of the nursing managers of each hospital and the supervision of the two graduate students. WeChat (a popular social networking tool in China) was utilized to conduct the electronic questionnaire. Those who chose the same option in questionnaires and those who missed >10% of items were excluded. After filtering, 1,038 questionnaires were included in the subsequent analysis with a response rate of 98.48%.

### Data Analysis

The analysis of the collected data was carried out using SPSS 26, Excel, and AMOS 23.0 software (both by IBM, Armonk, NY, USA). First, the demographic and work-related characteristics of the participants were determined using descriptive statistics. Pearson's correlation coefficients were used to calculate the correlation between all variables. Multivariable linear regression was used to predict work-related factors. Second, to test the validity and calculate the Cronbach alpha coefficient to estimate internal consistency, confirmatory factor analysis was conducted using AMOS. Third, to explore the link between the three emotional labor strategies, burnout, and presenteeism, the structural equation model was implemented and the mediating effects of job burnout were tested. The maximum likelihood method was used to confirm interrelationships and parameters between the variables in the structural equation modeling (SEM). We assessed the adequacy by the likelihood ratio (χ^2^/*df*), adjusted goodness of fit index (AGFI), Tucker–Lewis fit index (TLI), comparative fit index (CFI), standardized root mean square residual (SRMR), and root means the square error of approximation (RMSEA). An RMSEA <0.05, SRMR <0.08, and χ^2^/*df* < 3 indicated good model fit (44), while the other indices such as AGFI >0.90 can be construed as an acceptable fit (45).

### Measurements

#### Sociodemographic Characteristics

The demographic questionnaire included gender, age in years, marital status, professional title, employment status, monthly income (RMB, yuan), and weekly overtime. The criterion age was categorized as 20–30, 30–40, 40–50, and >50 in years. Marital status was categorized as unmarried, married without children, married with children. Professional title was categorized as either nurse, senior nurse, nurse supervisor, or above. Employment Status was categorized as either contractual or permanent. The biggest difference between contractual and permanent status is that the employer may have the right to discontinue the contract after the contract period has expired, while permanent employees work until retirement. Monthly Income (RMB, yuan) was categorized as <3,000 RMB, 3,000–5,000 RMB, >5,000 RMB. The number of times working overtime per week was categorized as 0, 1–2, 3–4, and >5.

#### Emotional Labor Strategies

The emotional labor strategy scale was used to assess the emotional performance strategies of the nurses in clinical work. As discussed above, the emotional labor strategy scale has two factors which are surface acting and deep acting (46). Based on the Emotional labor strategy Scale, the study of Luo combined the Chinese clinical nursing reality, revised by psychology and nursing professionals, and formed a questionnaire including 3 dimensions with 16 items (47). The Chinese version of the nurse emotional labor strategy scale consists of surface acting (SA, 7 items), deep acting (DP, 3 items), and emotionally expressed demands (EED, 4 items). Emotionally expressed demands refers to when nurses are required to show specific behaviors or expressions to reflect the image of the nurse to be established in the hospital. Each dimension was scored on a Likert-6 scale. In our study, the values of Cronbach alpha for three subscales were 0.85, 0.70, and 0.812, respectively. Higher scores indicate nurses experience higher levels of emotional labor.

#### Maslach Burnout Inventory

The 22-item self-report Maslach Burnout Inventory (MBI) was used to measure burnout, which consisted of emotional exhaustion (EE, 9 items, depersonalization (D, 5 items), and low personal accomplishment (LPA, 8 items) (24). MBI responses range from 0 (never) to 6 (every day). The nine-item emotional exhaustion subscale mainly evaluates emotional reactions caused by excessive work stress. The five-item depersonalization subscale mainly evaluates stress-induced attitudes and feelings toward the service recipient. The eight-item personal accomplishment subscale mainly describes stress-induced perceptions of the work of oneself. The scale has been established as a reliable and valid measurement in other studies (48–50). In this Study, the Chinese version of the MBI revised by Hua is used (51). Cronbach's alpha for three subscales were 0.9, 0.74, and 0.84, respectively.

#### Stanford Presenteeism Scale

The Chinese version of the 6-item Stanford Presenteeism Scale (52), produced by the study of Koopman et al. (53), was used to estimate health-related productivity loss. It is a tool for evaluating the loss of productivity or performance due to presenteeism caused by specific health problems (53). Responses for presenteeism ranged from 1 (completely disagree) to 5 (totally agree). Six items make up the SPS-6, containing two dimensions of finishing work (four items) and avoiding distraction (two items scored in reverse). The Cronbach's coefficient of the scale was 0.71 in this investigation.

## Results

### General Participants Characteristics

Demographic and work-related characteristics and scores of presenteeism among 1,038 nurses are shown in Table 1. Among the 1,038 participants, 97.5% were female. The average age was 31.2 ± 10.42 years. The number of married people with children (60.9%) was greater than unmarried ones (26.2%) and married ones without children (12.9%). Among the participants, senior nurses were the majority at 52.3%, and 48.7% of the participants worked overtime once or twice a week. In the case of employment status, contractual (91.5%) made up the majority. Nurses who earned 3,000–5,000 RMB were the majority at 60.4%.

**Table 1:** Demographic and working characteristics of nurses (*N* = 1,038) and scores of presenteeism.

**Variable**	**Category**	***N* (%)**	**Mean ± SD**
Gender	Female	1,012 (97.5)	14.18 ± 4.31
	Male	26 (2.5)	13.92 ± 5.11
Age (years)	20–30	598 (57.6)	13.88 ± 4.29
	30–40	367 (35.4)	14.60 ± 4.41
	40–50	67 (6.5)	14.37 ± 4.01
	>50	6 (0.6)	15.83 ± 4.88
Marital status	Unmarried	272 (26.2)	13.96 ± 4.31
	Married without children	134 (12.9)	13.88 ± 4.32
	Married with children	632 (60.9)	14.33 ± 4.33
Professional title	Nurse	249 (24.0)	14.13 ± 4.64
	Senior nurse	543 (52.3)	14.00 ± 4.28
	Nurse supervisor and above	246 (23.7)	14.61 ± 4.09
	Permanent	88 (8.5)	14.19 ± 4.32
Weekly overtime	0	341 (32.9)	13.53 ± 4.07
	1–2	506 (48.7)	14.06 ± 4.22
	3–4	140 (13.5)	15.51 ± 4.65
	>5	51 (4.9)	15.94 ± 4.88
Employment status	Contract	950 (91.5)	4.08 ± 4.42
	Permanent	88 (8.5)	14.19 ± 4.32
Monthly income	<3,000	92 (8.9)	13.58 ± 4.77
	3,000–5,000	627 (60.4)	14.30 ± 4.34
	>5,000	319 (30.7)	14.10 ± 4.15

### Correlations Analysis Among Emotional Labor, Burnout, and Presenteeism

Presenteeism was significantly positively correlated with surface acting, emotionally expressed demands, deep acting, emotion exhaustion, depersonalization, and low personal accomplishment (*P* < 0.01), according to correlation analysis. Notably, presenteeism had a significant negative association with deep acting (*P* < 0.01). Details can be found in Table 2.

**Table 2:** Means, standard deviations, and correlations for all variables.

**Variables**	**Mean ± SD**	**1**	**2**	**3**	**4**	**5**	**6**	**7**	**8**	**9**
SA	23.37 ± 6.84	1								
EED	13.41 ± 4.11	0.395^*^	1							
DA	14.60 ± 2.40	−0.019	0.179^*^	1						
EE	20.21 ± 11.42	0.477^*^	0.224^*^	−0.105^*^	1					
D	5.12 ± 5.27	0.467^*^	0.228^*^	−0.150^*^	0.658^*^	1				
LPA	15.15 ± 10.73	0.155^*^	−0.022	−0.384^*^	0.155^*^	0.224^*^	1			
Emotional labor	51.38 ± 9.72	0.865^*^	0.745^*^	0.309^*^	0.404^*^	0.388^*^	0.005	1		
Burnout	40.47 ± 20.39	0.453^*^	0.153^*^	−0.326^*^	0.784^*^	0.711^*^	0.726^*^	0.329^*^	1	
Presenteeism	14.18 ± 4.33	0.301^*^	0.212^*^	−0.139^*^	0.482^*^	0.364^*^	0.160^*^	0.442^*^	0.267^*^	1

*SA, surface acting; EED, emotionally expressed demands; DA, deep acting; D, depersonalization; EE, emotional exhaustion; LPA, low personal accomplishment*.

* *P <0.01*.

### Multi-Variable Linear Regression

The sociodemographic and job-related factors affecting presenteeism were predicted using multiple linear regression models. Presenteeism was explained by 22.8% of the variance in the final model as shown in Table 3 (*P* < 0.001). At the same time, more frequent weekly overtime and >50 years of age had predicted higher presenteeism in the regression model. The results also revealed that the higher the score of emotional labor and burnout, the more frequent the presenteeism.

**Table 3:** Results of multivariable linear regression to predict presenteeism factors.

**Variable**	**β**	***t***	***P***	***VIF***
**Gender (vs. male)**				
Female	0.034	1.222	0.222	1.049
**Age (vs. 20–30 years)**				
30–40	0.071	1.857	0.064	1.974
40–50	0.060	1.664	0.096	1.741
>50	0.046	2.067	0.039	1.112
**Marital status (vs. unmarried)**				
Married without children	0.004	0.139	0.890	1.367
Married with children	0.016	0.393	0.694	2.147
**Professional title (vs. nurse)**				
Senior nurse	0.005	0.144	0.886	1.960
Nurse supervisor and above	0.025	0.506	0.613	3.172
**Weekly overtime (vs.0)**				
1–2	0.062	1.780	0.075	1.273
3–4	0.157	4.628	0.000	1.220
>5	0.120	3.760	0.000	1.093
**Employment status (vs. permanent)**				
Contract	0.018	0.560	0.576	1.396
**Monthly income (vs**. ** <3,000**)				
3,000–5,000	0.064	1.281	0.201	1.396
>5,000	0.047	0.884	0.377	3.333
**Emotional labor**	0.214	7.063	0.000	1.154
**Burnout**	0.407	13.415	0.000	1.233

### Measurement Model and Structural Equation Model

**Figure 2:**
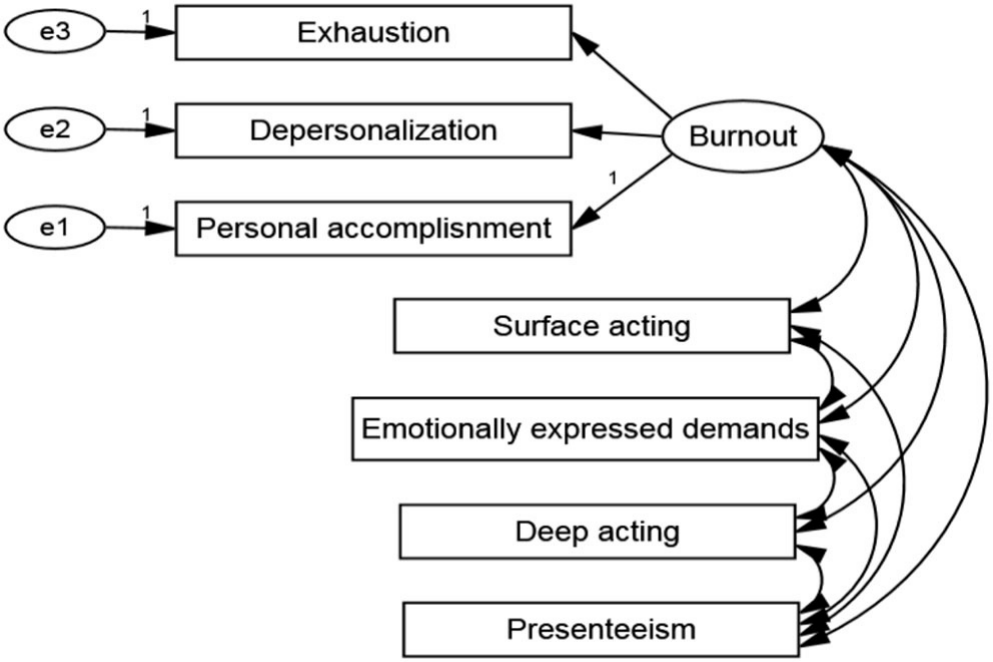
The measurement model.

**Figure 3:**
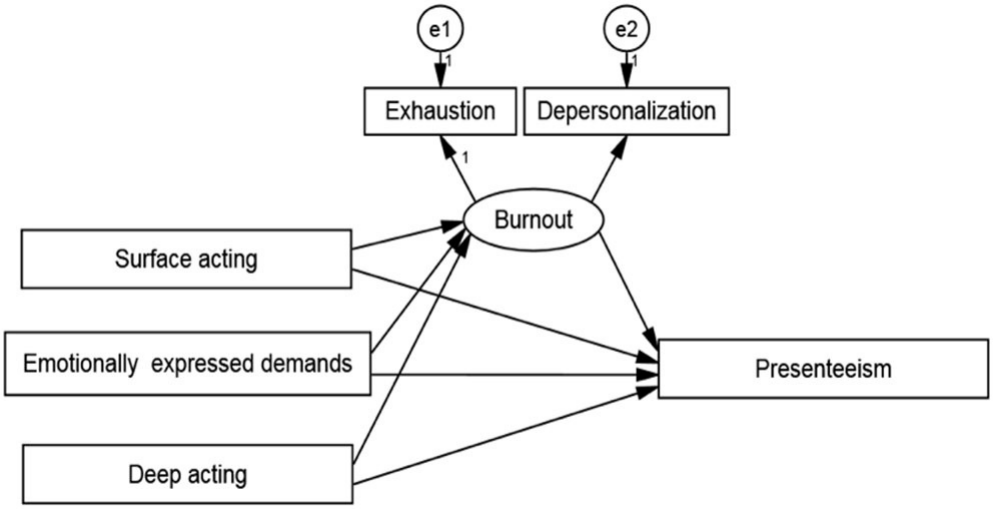
The structural equation model.

Pearson's correlation coefficient for presenteeism, burnout subscales, and emotional labor subscales was used to create a measurement model with four latent constructs and three observable variables. To assess the model fit using the maximum likelihood estimate, confirmatory factor analysis was performed. The initial fit indices of the measurement model had indicated that the factor loading of personal accomplishment (observed variables of burnout) was <0.5, thus this variable was eliminated. Figure 2 depicts the measuring model. The measurement model was used to construct the structural model (Figure 3). The modification indices were used to correct the structural model, and the fit indices showed that the structural model with good fit was *CMIN*/*df* = 1.371, RMSEA = 0.019, SRMR = 0.009, AGFI = 0.991, CFI = 0.999.

Table 4 displays the standardized estimates, critical ratio, standardizing effects, and mediating effect ratio for the route analysis. The direct impact on presenteeism was not significant (β = 0.033, *P* = 0.386), but surface acting had a considerable effect on burnout (β = 0.542, *P* < 0.001). On the other hand, surface acting had a substantial indirect influence on burnout (β = 0.204, *P* < 0.001). Burnout and presenteeism were significantly affected by emotionally expressed demands (β = 0.095, *P* = 0.004) and (β = 0.114, *P* = 0.001). Burnout and presenteeism were negatively linked with deep acting (β = −0.165, *P* = 0.001) and (β = −0.107, *P* = 0.001). The indirect effects of emotionally expressed demands and deep acting on presenteeism were 0.036 and −0.064, respectively.

**Table 4:** Standardized estimates, critical ratios, and standardized direct, indirect, and total effect and mediating effect ratio.

**Endogenous variable**	**Path**	**Exogenous variable**	**CR**	**Direct effect SE (*P*)**	**Indirect effect SE (*P*)**	**Total effect SE (*P*)**	**Mediating effect ratio (%)**
Burnout	←	Surface acting	15.121	0.542 (<0.001)		0.542 (<0.001)	
	←	Emotionally expressed demands	2.904	0.095 (0.004)		0.095 (0.004)	
	←	Deep acting	−5.474	−0.165 (<0.001)		−0.165 (<0.001)	
Presenteeism	←	Surface acting	2.269	0.033 (0.386)	0.204 (<0.001)	0.236 (<0.001)	86.44
	←	Emotionally expressed demands	3.59	0.114 (0.001)	0.036 (0.005)	0.150 (<0.001)	24.00
	←	Deep acting	−4.579	−0.107(<0.001)	−0.064 (<0.001)	−0.169 (<0.001)	63.31
Presenteeism	←	Burnout	10.367	0.438 (<0.001)		0.376 (<0.001)	

*←, Model pathway*.

## Discussion

In this study, 1,038 in-care nurses from six tertiary-level hospitals in Shaanxi Province were studied *via* constructing a structural equation model to investigate the correlations between three factors of emotional labor, burnout, and presenteeism. Deep acting alleviated burnout and reduced presenteeism directly, whereas emotionally expressed demands raised job burnout and presenteeism. Burnout played a completely mediating effect between surface acting and presenteeism.

First, the average presenteeism score was 14.18 (4.33). The SPS-6 score of China is greater than that of other countries (54). It may be related to the medical system of China. Nurses who work in public hospitals experienced more presenteeism (55). Health workers in China are extraordinarily overworked and have lower incomes than their counterparts in Europe and the United States (56). Multivariable linear regression carried out in this study has predicted demographic factors associated with presenteeism, and results revealed that higher presenteeism was predicted by more weekly overtime and >50 years of age. The results may be related to work stress, poor health, and organizational norms (55). It is noteworthy that presenteeism more likely occurred in older employees. Nurses with lengthy experience seemingly have a negative view of meeting workplace demands (8). Furthermore, nurses experience higher presenteeism with more frequent weekly over time, as previously reported (57). This suggested that managers should intervene to reduce presenteeism.

Second, this study examined the correlations between the research variables among the nurses. The Pearson's correlation coefficients for surface acting, emotionally expressed demands, emotion exhaustion, depersonalization, low personal accomplishment, and presenteeism were all significant. This study identified that deep acting and emotionally expressed demands directly and indirectly impacted the presenteeism of the nurses. Deep acting created an internal-external union that might result in favorable psychological states, which could expend limited resources on internalizing emotional expression and inner feeling (29, 58, 59). On the other hand, acting in good faith (deep acting) negatively affected presenteeism, congruent with a previous study (60). Therefore, nurse managers should be aware that deep acting emotion strategies could effectively reduce presenteeism. To improve the emotion management skills of the nurses, various research had indicated that self-training mindfulness, emotion therapy, empathy training, and educational training interventions can improve emotion regulation (61, 62). These interventions include self-emotional management, hospital management of caregivers, and organizational management. Emotion expression demands were also linked to greater burnout and presenteeism according to the study. It is difficult to compare our results to previous research because there are few studies in which emotionally expressed demands could predict presenteeism. The emotionally expressed demands are the particular behavior or emotion which mirrors the image of the nurse the hospital wishes to present. In turn, nurses remain courteous despite the patients make unreasonable demands (47). Undoubtedly, the requirements of the nursing profession had placed high-work demands on nurses. When nurses were constantly subjected to high expectations (for example, severe workload and emotional dissonance), they were positively linked to presenteeism (63). It should be noted that there is a lack of research indicating a link showing how emotionally expressed demands are related to presenteeism. Further study on this topic is necessary.

Surface acting did not directly influence presenteeism, which did not correspond with earlier research (30). The reason for this might be that, to our knowledge, this is the first study to construct a structural equation model to show the path association between the three emotional labor methods, burnout, and presenteeism in nurses. Furthermore, future studies should account for the confounding variables to explore the correlation between variables in this study.

In this study, we anticipated that burnout could mediate the effect of emotional labor techniques on presenteeism, and SEM has clarified this relationship. The structural equation model indicated that burnout has partially mediated the relationship between deep acting, emotionally expressed demands, and presenteeism, with a mediating effect of 24 and 63.31% of the total impact. Additional data also confirmed that job burnout played a complete mediating role in surface acting and presenteeism, with a mediating effect of 86.44%. The risk of nurse emotional dissonance and lost productivity can be mitigated by relieving nurse burnout. A previous study revealed that nurses are required to display a particular expression at work that matches the requirements of the organization by expending resources according to the conservation of resources theory (64). The workload was also positively associated with presenteeism through higher burnout levels (36). Emotional dissonance caused by long-term surface acting and matching emotion expression demands will undoubtedly increase the emotional resources of the nurses, which only disappears after a lengthy recovery period. However, nurses must continue to work to meet high job demands, which disrupts the restoration process, causes chronic exhaustion, and leads to productivity loss. Given the shortage of nurses and presenteeism, hospital administrators should not force nurses to express the emotional labor required by the hospital in the face of rude or demanding patients (65). Burnout has been linked to dissatisfaction, missed care, self-compassion, and occupational turnover intention, according to studies (48, 66–68). Furthermore, interventions to improve the ability of the nurses to regulate emotions in specific patient settings may prevent burnout and productivity loss.

Nevertheless, several limitations should be acknowledged. First, cross-sectional research resulted in insufficient data compared to a longitudinal design, which have drawn a causal association between emotional labor, burnout, and presenteeism. A subsequent longitudinal study will still have to evaluate the findings, and such linkages to inductive reasoning change the correlations between these variables. Secondly, our research participants comprised nurses from six hospitals but failed to consider the distinctions across the departments, such as in emergency, surgery, and critical care. In addition, the majority of research participants were women. Thus, further studies should analyze the disparities between nurse populations and gender. Third, only nurses from six hospitals in Xi'an city were chosen using convenience sampling. Although the representativeness of the sample was restricted, the structural equation model might be employed in future research to investigate the link between these variables using random sampling.

## Conclusion

After investigating the demographic and job-related factors to predict presenteeism, we discovered that nurses who worked more overtime each week and were >50 years old were more likely to exhibit presenteeism. The nursing managers should reasonably arrange human resources, adjust shifts, reduce overtime work, and focus on nurses with rich experience. Different factors of emotional labor have different effects on presenteeism, according to the findings. Self-training mindfulness, emotion therapy, or other effective methods could improve the emotional management skills of the nurses and help them master the correct emotional labor strategies. The structural equation model verified our prediction that burnout acted as a mediator between diverse emotional labor techniques and presenteeism. Improving emotional management skills and alleviating burnout can reduce presenteeism. Nursing administrators should focus on alleviating the emotional exhaustion of nurses by reducing workload and increasing support among colleagues, among other methods.

## Data Availability Statement

The raw data supporting the conclusions of this article will be made available by the authors, without undue reservation.

## Ethics Statement

The studies involving human participants were reviewed and approved by Affiliated Hospital of Shaanxi University of Traditional Chinese Medicine, Shaanxi Province, China. The patients/participants provided their written informed consent to participate in this study.

## Author Contributions

JS implemented this study and was responsible for data collection and analysis and writing. XL supported the investigation and data analysis. ZQ and RZ provided assistance in reviewing the manuscript. FL guided the study design and interpretations. All authors approved the final paper.

## Funding

The Shaanxi Provincial Department of Education 2018 Annual Special Scientific Research Program funded the study (No. 18JK0201).

## Conflict of Interest

The authors declare that the research was conducted in the absence of any commercial or financial relationships that could be construed as a potential conflict of interest.

## Publisher's Note

All claims expressed in this article are solely those of the authors and do not necessarily represent those of their affiliated organizations, or those of the publisher, the editors and the reviewers. Any product that may be evaluated in this article, or claim that may be made by its manufacturer, is not guaranteed or endorsed by the publisher.
